# The Default Mode Network and the Working Memory Network Are Not Anti-Correlated during All Phases of a Working Memory Task

**DOI:** 10.1371/journal.pone.0123354

**Published:** 2015-04-07

**Authors:** Tommaso Piccoli, Giancarlo Valente, David E. J. Linden, Marta Re, Fabrizio Esposito, Alexander T. Sack, Francesco Di Salle

**Affiliations:** 1 Department of Cognitive Neuroscience, Faculty of Psychology and Neuroscience, University of Maastricht, Maastricht, The Netherlands; 2 Department of Biomedicine and Clinical Neuroscience, University of Palermo, Palermo, Italy; 3 MRC Centre for Neuropsychiatric Genetics and Genomics, Institute of Psychological, Medicine and Clinical Neurosciences, Cardiff University School of Medicine, Cardiff, United Kingdom; 4 Department of Electronics, Information and Bioengineering, Politecnico di Milano, Milano, Italy; 5 Department of Medicine and Surgery, University of Salerno, Salerno, Italy; Yale University, UNITED STATES

## Abstract

**Introduction:**

The default mode network and the working memory network are known to be anti-correlated during sustained cognitive processing, in a load-dependent manner. We hypothesized that functional connectivity among nodes of the two networks could be dynamically modulated by task phases across time.

**Methods:**

To address the dynamic links between default mode network and the working memory network, we used a delayed visuo-spatial working memory paradigm, which allowed us to separate three different phases of working memory (encoding, maintenance, and retrieval), and analyzed the functional connectivity during each phase within and between the default mode network and the working memory network networks.

**Results:**

We found that the two networks are anti-correlated only during the maintenance phase of working memory, i.e. when attention is focused on a memorized stimulus in the absence of external input. Conversely, during the encoding and retrieval phases, when the external stimulation is present, the default mode network is positively coupled with the working memory network, suggesting the existence of a dynamically switching of functional connectivity between “task-positive” and “task-negative” brain networks.

**Conclusions:**

Our results demonstrate that the well-established dichotomy of the human brain (anti-correlated networks during rest and balanced activation-deactivation during cognition) has a more nuanced organization than previously thought and engages in different patterns of correlation and anti-correlation during specific sub-phases of a cognitive task. This nuanced organization reinforces the hypothesis of a direct involvement of the default mode network in cognitive functions, as represented by a dynamic rather than static interaction with specific task-positive networks, such as the working memory network.

## Introduction

Cognitive functions arise from the orchestrated activation and cooperation of networks of regions whose specific relationship varies dynamically across functional states [1, 2]. The default mode network (DMN), defined as being related to a baseline cognitive state, is involved in large-scale brain organization, both during rest and cognitive tasks [3–5]. The DMN has been identified through the observation of its deactivation across a range of cognitive tasks [6,7] and further refined through the analysis of coherent patterns of low frequency fMRI signal fluctuations [8–10].

The DMN commonly comprises the medial prefrontal cortex (MPFC), the posterior cingulate/retrosplenial cortex (PCC/Rsp), and the inferior parietal lobule (IPL) [2]. Although it has been suggested repeatedly that the DMN is generally related to the working memory network (WMN) [5,11–15] and thus potentially involved in the neural mechanism underlying working memory [16,17], the process-dependent intimate link between the DMN and the WMN has not been clarified. In fact, most of the above mentioned results were obtained by using an N-back task [18], a commonly used working memory task which does not allow one to dynamically separate the three fundamental working memory sub-processes, called encoding, maintenance, and retrieval, as these temporally overlap across consecutive N-back trials [11].

Recent evidences have highlighted the involvement of some DMN regions during both other working memory tasks and episodic memory tasks [16,19–22], suggesting that DMN nodes could be activated differently during distinct memory phases. However, this aspect cannot be explained with the general concept of a DMN task-related global deactivation, but requires a more complex functional relationship between networks to be addressed. This study aimed to concretely address this aspect in a parametric working-memory fMRI connectivity study.

We hypothesized that the functional connectivity between the networks supporting working memory change dynamically across the various cognitive phases. Each of these phases is in fact characterized by complex cognitive engagement of multiple brain regions [23–26]. In our task, it was possible to model the temporal progression of working memory processing across three consecutive phases: (i) encoding of the information, (ii) maintenance of the information, (iii) retrieval of the information for response selection. We here used a delayed working memory spatial paradigm [27] and studied the functional connectivity within and between the WMN and the DMN nodes during each of the three phases. We were, therefore, able to systematically assess whether intra- and/or inter-network functional connectivity depended on working memory phase. We then expected changes in the role of the DMN depending on phase of task execution, by means of a modulated cross-network correlation between DMN and WMN.

## Methods

Fourteen healthy, subjects (8 males, age range 20–30) were recruited for this study. All of them had no history of neurological or psychiatric disorders, normal or corrected to normal visual acuity. All subjects were right-handed according to the Edinburgh Questionnaire [28].

Ethical Committee of the Faculty of Psychology at the University of Maastricht approved the study. All the subjects gave written, informed consent, in accordance with the principles expressed in the Declaration of Helsinki.

### Working memory task

**Fig 1:**
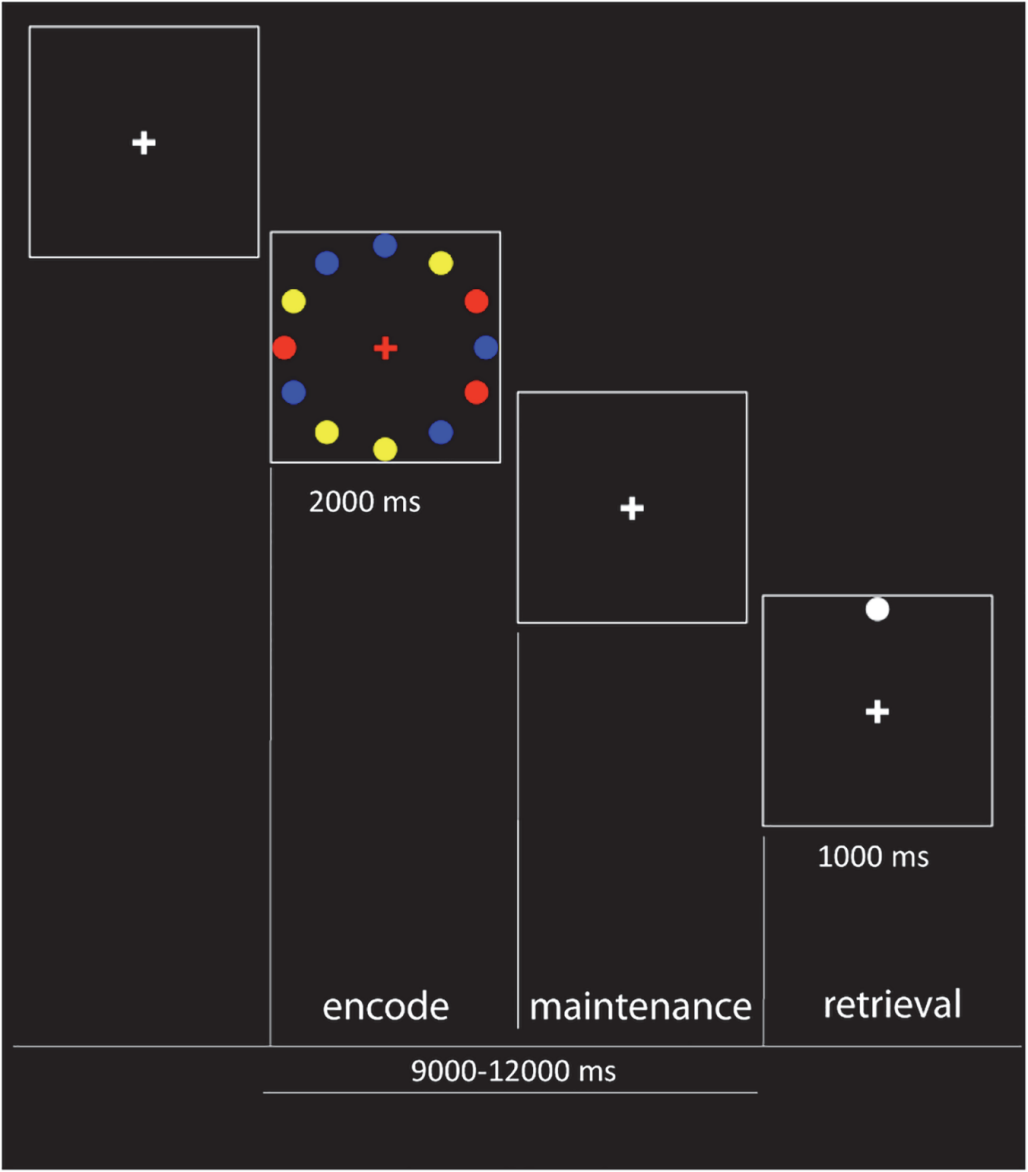
Working memory delayed spatial working memory task.

All subjects performed a delayed, location-based, spatial working memory paradigm, which builds on the work by Mottaghy [27]. In this task, the subjects were asked to judge whether a given target stimulus had been part of a previous memory stimulus set or not. The memory set consisted of one, three, or five circles (corresponding to three load conditions) of equal diameter, presented for 2000 ms. The circles were randomly presented at twelve possible locations along a circumference and in three different colors: blue, yellow or red. To avoid physical presentation differences across load conditions, each memory stimulus contained both the circles of the present load condition, and (in different colors) as many circles as needed to cover the twelve positions. The central fixation cross was white and randomly assumed one of the three colors, indicating which set of circles to focus on. After the stimulus offset, the cross assumed the white color again. The spatial stimulus was followed by a delay period jittered between 9 and 12 s from the onset of each display to the subsequent target stimulus. The target stimulus consisted of one white circle, randomly located in one of the twelve positions. Subjects were asked to respond with the right (‘yes’) or left (‘no’) index fingers pressing two different keypads, deciding whether the target stimulus had been part of the previous spatial stimulus, or not. ‘Yes’ and ‘no’ trials were pseudo-randomized to be equal in number. A resting period of 12 s lasted from the button press to the following memory stimulus onset. Subjects were asked to fixate the white cross during the whole experiment. A psychophysical pilot study revealed a clear and reliable working memory load effect across conditions, confirming the suitability of this task. Each subject was trained on the task for 15 minutes the day before the fMRI data acquisition. The experiment consisted of 90 working memory trials, 30 per load, divided into three functional runs (Fig 1).

### Behavioral output

Working memory performance was calculated using reaction times (elapsed time between the presentation of the stimulus and the response) and accuracy (percentage of correct responses) as behavioral outcome. Only trials followed by correct response were considered for all the analyses. We calculated one-way repeated measures ANOVA using accuracy and reaction times as factors.

### Data acquisition

fMRI data were acquired on a Siemens Allegra 3T scanner. For the functional scan, T2*-weighted echo planar images (EPI) were acquired (TR 1500 ms, TE 30 ms, voxel 3.5x3.5x3.5 mm, flip angle 90°, 15’each run). Anatomical images were acquired using a magnetization-prepared rapid gradient echo (MP-RAGE) 3D T1-weighted sequence.

### Preprocessing of fMRI data

Image preprocessing was performed in BrainVoyager QX (Brain Innovation, Maastricht, The Netherlands). Functional image time-series was corrected for the differences in slice acquisition times, realigned with T1 volumes, and warped into the standard space of Talairach and Tournoux [29]. The resulting voxel-time-series were filtered in time and space. A high-pass temporal filter (7 cycles) was applied and a spatial smoothing, with an isotropic Gaussian kernel of 4 mm full width at half-maximum, was performed. For display purposes on the volumetric anatomy, individual maps were projected on the average normalized volumetric image. No additional pre-processing, such as whole-brain signal regression, was performed.

### Group analysis

#### Activations

A multi-subject random-effects general linear model (GLM) analysis was performed including all WMN runs.

We modeled each working memory trial with three predictors; encoding, starting at stimulus onset; maintenance, starting 4 fMRI volumes after the encoding, when the subject maintained the target positions in the working memory; and retrieval, from target stimulus onset, when the subjects had to perform a judgment. Each predictor had the duration of one functional volume (i.e., 1500 ms), to ensure that all the predictors had the same amplitude. BOLD responses were modeled by convolving each predictor with a canonical hemodynamic response. In the univariate statistical analysis, we therefore used nine predictors, modeling the three phases of the trial for the three loads separately plus an additional predictor to model trials with errors, for a total of 10 predictors.

We used the main effects map (thresholded using False Discovery Rate with q = 0.05) to identify de-activated regions within the DMN (MPFC, PCC/Rsp, left and right IPL) [2] and task-related activations within the WMN (DLPFC and IPS bilaterally) [1]. As a post-hoc analysis, we then considered event-related averages (i.e. the average across all the repetitions of a given trial) of each of these regions in order to inspect the modulation of the hemodynamic responses by the load.

### Single subject analysis

Connectivity analyses were performed at a single subject level, the subsequent statistical assessment was done via a second level analysis. For each subject, we randomly selected one of the three functional runs to identify the nodes, and used the remaining two runs to evaluate connectivity profiles. This procedure ensured independence between the region selection procedure and the connectivity evaluation, ruling out possible biases due to data re-usage.

#### Region of Interest selection

For each subject, we randomly selected one out of the three runs to localize ROIs, and used the remaining two runs to conduct the connectivity analyses (localizing run), selecting the nodes of interest by means of a single-subject GLM, overall F-statistic on the main effect (FDR, q = 0.05). This procedure led to the identification of a set of task-positive and a set of task-negative areas. From the task-positive map, we selected four ROIs relating to the working memory task: left and right DLPFC and left and right IPS. From the task-negative map, we selected four ROIs relating to the DMN: MPFC, PCC/Rsp, left and right IPL [2]. These areas could be consistently identified in all the subjects. Inferior parietal cortex and DLPFC were chosen based on their high degree of consistency across the fMRI studies involving working memory [1,30]. Furthermore, in the context of the DLPFC, the peaks of activation were located in Brodmann area 9 for all subjects (S1 Table). As a control analysis, we also selected two ROIs in auditory cortex (right and left, see S2 Table) and used them as control region, as we do not expect any modulation of connectivity between these areas and the regions investigated in this study.

#### Activations analysis

We calculated a ROI GLM analysis from single subjects’ ROIs, using the three phases of the task (encode, maintenance, and retrieval) as conditions, to test the activity of each region in the different phase of the cognitive process against baseline. In this analysis we used the same ROIs as in the connectivity analysis (see below). We also computed a two-way repeated measures ANOVA using load and phase as within subjects factor (FDR, q = 0.05).

#### Functional connectivity analysis

In the connectivity analysis, we used the remaining two runs (connectivity runs) and constructed a cube of maximally 27 voxels centered on the peaks of the F-map in the DMN and WMN nodes, as estimated from the localizing run. Voxels within the cube but outside the cortex were excluded from the analysis. The time series within these cubes were averaged and the resulting time series (one per region) were analyzed using Beta Series Correlation (BSC) approach [31], which has been successfully employed in a large number of studies [32–34]. In BSC, regional time-courses are modeled with a set of regressors with each predictor coding for a specific event. In our design, we modeled each working memory trial with three predictors, (one for each phase of the task, convolving with an estimate of the hemodynamic response function, similarly to what described in the univariate analysis), resulting in maximally 60*3 predictors for the two connectivity runs, plus a predictor accounting for error trials (when present). The patterns of temporal variation across the experiment of the regression coefficients (i.e., betas) are then compared across different areas using a Pearson correlation. This approach is different from conventional resting state connectivity, which characterizes connectivity by means of temporal correlation between raw BOLD time series of different voxels; BSC first summarizes each trial with a set of coefficients describing the amplitude in different phases of the trial, and then investigates the temporal correlation of task-modulated temporal variations of response amplitude. With such an approach, it is possible to study differences in connectivity across different experimental conditions and, most importantly, modulations of connectivity across different stages of the same task. We used a 1500 ms duration for each predictor; however, duration was not a critical factor since correlation was calculated for each phase separately. To ensure that changes of overall signal intensity and variance across runs did not affect the connectivity measures, we standardized the Beta Series of each run with a z-score transformation.

The group analysis was performed with a second level analysis (random effects): we first computed the BSC for each subject, and then used subject’s estimates as input to a subsequent analysis. For each of the three conditions and for each of the three loads, we computed the pairwise Pearson correlation between the beta series of two distinct areas, and repeated this with all the possible combinations. This analysis resulted in 9 correlation values per connection, for a total of 28 connections. It is worth noting that, since the correlations are calculated for each experimental condition and load separately (e.g., encoding, load 1), we do not expect their values to be influenced by univariate load effects in one or in both of the areas compared. Moreover, since the separation between the predictors is larger than 4 seconds, the different conditions can be identified [31]. Before conducting the group analysis, we transformed the correlation values with a Fisher transformation, to ensure normally distributed observations [35]. The group analysis consisted of a repeated measure two-way ANOVA with task phase and load as factors for each connection. The results obtained were then corrected for multiple comparisons (across the different connections and test, 9*28 tests) using False Discovery Rate (FDR) [36].

## Results

### Performance

**Fig 2:**
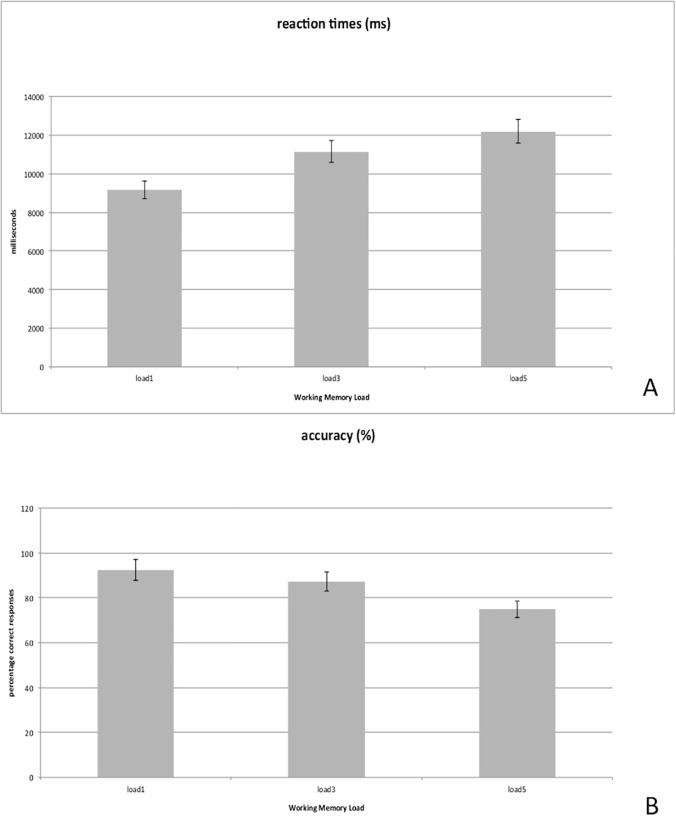
Behavioral output. Charts A and B show that accuracy (percentage of correct responses) increases while reaction times (measured from the onset of the target stimulus to the subject's response) decrease at higher cognitive loads. Error bars indicate SD.

Fig 2 shows the behavioral data. Reaction times increased and accuracy decreased with working memory load.

### Activations

**Fig 3:**
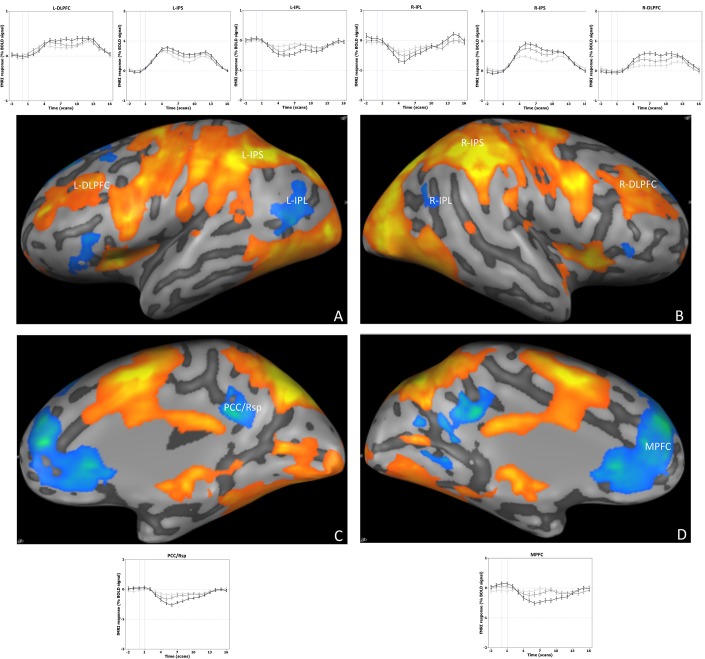
Multi-subject, whole brain random-effects GLM maps, thresholded at (q)FDR = 0.05. IPS = intra-parietal sulcus; DLPFC = dorso-lateral prefrontal cortex; MPFC = medial prefrontal cortex; PCC/Rsp = posterior cingulate/retrosplenial cortex; IPL = inferior parietal lobule. R- = right; L- = left. Graphs show event related average time-course relative to each ROI.

The main effects map, obtained from a multi-subject random-effects general linear model (GLM) analysis, included both de-activated regions (DMN) and task-related activations (WMN). When we performed the post hoc event related average analysis, we found a higher activation at higher working memory loads in the task-related regions and higher de-activation at higher loads in DMN regions, thus confirming the model of two anti-correlated networks (Fig 3). These findings were consistent across individual subject GLMs. Single subject regions of interest (ROIs) GLM analysis showed a highly significant activation in all the working memory regions and a deactivation in DMN regions in all of the task conditions (Table 1). Two-way repeated measures ANOVA with load and phase as within subjects factor (FDR, q = 0.05) showed that there was a significant phase effect in each region of both networks with the exception of right IPL whereas the load effect was limited to the right IPL in the DMN and the right and left intra-parietal sulcus (IPS) in the WMN (Table 2). This analysis confirmed that the areas that were selected from the individual localizer runs also showed task-related activity in the independent runs from the same participants.

**Table 1:** Across-subjects region of interest activations analysis (general linear model).

Networks	Brain regions	ENCODE		MAINTENANCE		RETRIEVAL	
		p	t	p	t	p	t
*Default Mode Network*	**PCC/Rsp**	7,E-04*	-7,415	8,E-05*	-5,643	1,E-05*	-6,615
	**MPFC**	8,E-04*	-4,366	1,E-03*	-4,136	3,E-04*	-4,838
	**Right IPL**	2,E-05*	-6,517	7,E-06*	-5,686	1,E-03*	-3,89
	**Left IPL**	6,E-05*	-7,257	1,E-03*	-3,992	1,E-05*	-6,834
*Working Memory Network*	**Right IPS**	2,E-09*	14,524	1,E-05*	6,609	1,E-08*	12,15
	**Left IPS**	2,E-05*	14,143	1,E-08*	12,601	1,E-07*	10,407
	**Right DLPFC**	4,E-05*	6,081	1,E-05*	6,587	2,E-09*	12,042
	**Left DLPFC**	7,E-06*	7,17	8,E-06*	7,096	1,E-06*	9,031

PCC/Rsp: posterior cingulate/retrosplenial cortex; MPFC: medial prefrontal cortex; IPL: inferior parietal lobule; IPS: intra-parietal sulcus; DLPFC: dorso-lateral prefrontal cortex.

* = p values exceeding FDR threshold (q = 0.05).

**Table 2:** Modulation of ROIs activations: Across-subjects two-way repeated measures ANOVA with load and phase as within subjects factor.

Networks	Brain regions	PHASE	LOAD	INTERACTION
		p	p	p
*Default Mode Network*	**PCC/Rsp**	0,0002*	0,009	0,362
	**MPFC**	0,002*	0,774	0,050
	**Right IPL**	0,011	0,0004*	0,036
	**Left IPL**	0,002*	0,009	0,612
*Working Memory Network*	**Right IPS**	0,000001*	0,00001*	0,000004*
	**Left IPS**	0,000001*	0,0001*	0.0001*
	**Right DLPFC**	0,00003*	0,050	0,007*
	**Left DLPFC**	0,0005*	0,040	0,014*

PCC/Rsp: posterior cingulate/retrosplenial cortex; MPFC: medial prefrontal cortex; IPL: inferior parietal lobule; IPS: intra-parietal sulcus; DLPFC: dorso-lateral prefrontal cortex.

* = p values exceeding FDR threshold (q = 0.05).

### Connectivity

For each pair of ROIs, we considered the trial-by-trial correlation of the beta series, calculating Pearson correlation separately for each phase and load. This resulted in 9 different values per pair of ROIs, per subject, which were transformed with a Fisher transform to ensure normality. For each ROI pair, we performed a repeated measures two-way ANOVA, with phase and load as factors. We corrected for multiple comparisons using FDR with q = 0.05.

#### Effects of load

We found a load effect in functional connectivity within the WMN, in particular between right IPS and left IPS (p = 0.001) and between right IPS and left dorso-lateral prefrontal cortex (DLPFC) (p = 0.001). We did not find any load effect either within the DMN or between the two networks (Table 3).

**Table 3:** Modulation of functional connectivity between pairs of ROIs.

Networks	PAIRS	PHASE	LOAD	INTERACTIONS
		p	p	p
*Default Mode Network*	**PCC/Rsp—MPFC**	0,246	0,93	0,409
	**PCC/Rsp—Right IPL**	0,386	0,62	0,255
	**PCC/Rsp—Left IPL**	0,64	0,686	0,28
	**MPFC—Right IPL**	0,55	0,774	0,47
	**MPFC—Left IPL**	0,343	0,908	0,515
	**Right IPL—Left IPL**	0,571	0,018	0,237
*Working Memory Network*	**Right IPS—Left IPS**	0,331	0,0004*	0,225
	**Right IPS—Right DLPFC**	0,708	0,064	0,645
	**Right IPS—Left DLPFC**	0,699	0,0002*	0,958
	**Left IPS—Right DLPFC**	0,915	0,026	0,68
	**Left IPS—Left DLPFC**	0,129	0,007	0,424
	**Right DLPFC—Left DLPFC**	0,585	0,007	0,253
*Default Mode Network Vs Working Memory Network*	**PCC/Rsp—Right IPS**	0,011	0,399	0,815
	**PCC/Rsp—Left IPS**	0,013	0,274	0,076
	**PCC/Rsp—Right DLPFC**	0,0003*	0,326	0,389
	**PCC/Rsp—Left DLPFC**	0,001*	0,057	0,272
	**MPFC—Right IPS**	0,028	0,705	0,805
	**MPFC—Left IPS**	0,03	0,564	0,785
	**MPFC—Right DLPFC**	0,044	0,909	0,621
	**MPFC—Left DLPFC**	0,055	0,179	0,495
	**Right IPL—Right IPS**	0,004*	0,48	0,364
	**Right IPL—Left IPS**	0,013	0,696	0,307
	**Right IPL—Right DLPFC**	0,036	0,711	0,553
	**Right IPL—Left DLPFC**	0,007	0,588	0,364
	**Left IPL—Right IPS**	0,177	0,426	0,479
	**Left IPL—Left IPS**	0,026	0,745	0,237
	**Left IPL—Right DLPFC**	0,01	0,328	0,283
	**Left IPL—Left DLPFC**	0,005*	0,744	0,092

Across-subjects two-way repeated measures ANOVA with load and phase as within subjects factor (p values).

PCC/Rsp: posterior cingulate/retrosplenial cortex; MPFC: medial prefrontal cortex; IPL: inferior parietal lobule; IPS: intra-parietal sulcus; DLPFC: dorso-lateral prefrontal cortex.

* = p values exceeding FDR threshold (q = 0.*05)*.

#### Effects of phase

When we calculated the functional connectivity within each network, we did not find any modulation dependent on task phase. When we calculated the functional connectivity between the networks, we found a significant phase modulation for the following connections: PCC/Rsp with right DLPFC (p = 0.0001), PCC/Rsp with left DLPFC (p = 0.001), right IPL with right IPS (p = 0.004), and left IPL with left DLPFC (p = 0.005; Table 3). Apart from the connection between right IPL and right IPS, all of them were higher during encoding and retrieval than during maintenance. The connection between the two right parietal nodes (IPS and IPL) of the networks increases its strength through the task (Table 3).

We also calculated the mean functional connectivity in each phase of the task and found that all the pairs within each network were positively connected in all the three phases of the task (p = 0.000; Table 4). Conversely, the correlation between DMN and WMN differed substantially depending on the task phase. During the encoding phase, only one significant correlation was present: a positive functional connectivity link between left IPL and left DLPFC (p = 0.006). During the maintenance phase, we found a negative correlation of PCC/Rsp with right (p = 0.008) and left (p = 0.017) IPS, PCC/Rsp with right (p = 0.001) and left (p = 0.009) DLPFC, MPFC with right (p = 0.005) and left (p = 0.003) IPS, and MPFC with right DLPFC (p = 0.000). During the retrieval phase, there was a positive correlation of MPFC with left DLPFC (p = 0.017), right IPL with right IPS (p = 0.001), right IPL with left DLPFC (p = 0.012) and left IPL with left DLPFC (p = 0.005; Table 4). All these correlations survived correction with the false discovery rate.

**Table 4:** Across-subjects mean functional connectivity, in distinct phases of the working memory task.

Networks	PAIRS	ENCODE		MAINTENANCE		RETRIEVAL	
		p	t	p	t	p	t
*Default Mode Network*	**PCC/Rsp—MPFC**	2E-14*	11,462	2E-19*	16,086	6E-13*	10,289
	**PCC/Rsp—Right IPL**	1E-12*	10,118	2E-11*	9,110	7E-12*	9,441
	**PCC/Rsp—Left IPL**	3E-13*	10,508	1E-12*	10,064	2E-14*	11,504
	**MPFC—Right IPL**	2E-07*	6,234	3E-12*	9,751	7E-10*	7,942
	**MPFC—Left IPL**	1E-10*	8,441	5E-12*	9,544	1E-12*	9,896
	**Right IPL—Left IPL**	9E-17*	13,569	3E-18*	14,995	3E-20*	17,030
*Working Memory Network*	**Right IPS—Left IPS**	6E-21*	17,839	5E-21*	17,932	6E-18*	14,652
	**Right IPS—Right DLPFC**	2E-16*	13,116	1E-14*	11,709	6E-18*	14,680
	**Right IPS—Left DLPFC**	1E-14*	11,756	8E-14*	11,008	2E-12*	9,881
	**Left IPS—Right DLPFC**	7E-17*	13,644	5E-16*	12,883	6E-15*	11,937
	**Left IPS—Left DLPFC**	5E-16*	12,519	3E-18*	15,245	2E-16*	13,139
	**Right DLPFC—Left DLPFC**	4E-15*	12,084	4E-18*	14,787	5E-17*	13,785
*Default Mode Network Vs Working Memory Network*	**PCC/Rsp—Right IPS**	0,336	-0,973	0,008*	-2,792	0,185	1,347
	**PCC/Rsp—Left IPS**	0,994	0,008	0,017*	-2,487	0,185	1,349
	**PCC/Rsp—Right DLPFC**	0,481	-0,711	0,001*	-3,619	0,13	1,545
	**PCC/Rsp—Left DLPFC**	0,955	0,057	0,009*	-2,727	0,082	1,782
	**MPFC—Right IPS**	0,049	-2,027	0,005*	-2,954	0,955	-0,057
	**MPFC—Left IPS**	0,646	-0,463	0,003*	-3,150	0,982	-0,023
	**MPFC—Right DLPFC**	0,189	-1,335	0,0001*	-4,110	0,625	0,493
	**MPFC—Left DLPFC**	0,547	-0,607	0,095	-1,707	0,017*	2,487
	**Right IPL—Right IPS**	0,696	0,393	0,32	1,006	0,001*	3,645
	**Right IPL—Left IPS**	0,058	1,952	0,82	-0,229	0,007*	2,867
	**Right IPL—Right DLPFC**	0,395	0,861	0,635	-0,479	0,065	1,893
	**Right IPL—Left DLPFC**	0,035	2,178	0,485	-0,705	0,012*	2,617
	**Left IPL—Right IPS**	0,66	0,443	0,265	-1,131	0,258	1,147
	**Left IPL—Left IPS**	0,132	1,537	0,097	-1,698	0,101	1,676
	**Left IPL—Right DLPFC**	0,5	0,681	0,017*	-2,478	0,409	0,834
	**Left IPL—Left DLPFC**	0,006*	2,863	0,243	-1,184	0,005*	3,006

PCC/Rsp: posterior cingulate/retrosplenial cortex; MPFC: medial prefrontal cortex; IPL: inferior parietal lobule; IPS: intra-parietal sulcus; DLPFC: dorso-lateral prefrontal cortex.

*p = values exceeding FDR threshold (q = 0.05).

Combining the above results (modulation of connectivity across phases, and mean functional connectivity in each phase of the task), we found during encoding and retrieval a positive connectivity of IPL with DLPFC in the left hemisphere and, during retrieval, a positive connectivity of IPL and IPS in the right hemisphere. Conversely, during maintenance we found a negative connectivity between PCC/Rsp and DLPFC in both hemispheres.

In the control analysis, after correction with false discovery rate, we did not find any significant modulation of correlation between the control ROIs regions in the right and left auditory cortex and all the other ROIs (S3 Table).

## Discussion

**Fig 4:**
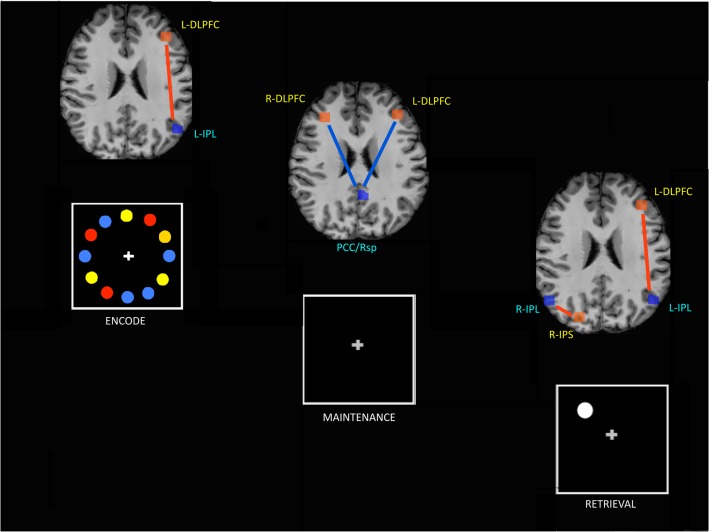
Functional connectivity between the WM network and the DMN in distinct phases of a working memory task. DLPFC: dorsolateral prefrontal cortex; IPS: intra-parietal sulcus; IPL: inferior parietal lobule; PCC/Rsp = posterior cingulate/retrosplenial cortex. Yellow fonts indicate WMN regions; blue fonts indicate DMN regions. Red lines: positive correlations; blue lines: negative correlations.

The current study aimed to investigate dynamic connectivity changes within as well as between the DMN and WMN, depending on the specific cognitive sub-phase during a behaviorally controlled working memory paradigm. This approach was aimed at more specifically addressing the question of the role of the different brain areas during working memory. In particular, we were interested in the functional connectivity between task-positive (WMN) and task-negative (DMN) networks. Interestingly, we did not find any phase modulation of functional connectivity within each network. Instead, we found that functional connectivity between certain regions of DMN and WMN is modulated by the working memory task phases (Table 3). Specifically, we found a positive correlation between the DMN and the WMN during the encoding and retrieval phases of working memory. These positive inter-network correlations between DMN and WMN are located in the left hemisphere, between DMN parietal and WMN prefrontal regions, with an additional positive correlation between right parietal regions of both networks during the retrieval phase. In contrast, during the maintenance phase, an anti-correlation between the core of DMN (PCC/Rsp) and prefrontal regions of the WMN is present bilaterally (Fig 4). Our findings suggest that the coupling between DMN and WMN is dynamic across the various phases of a working memory task and does not involve to the two networks as a whole, but engages different couples of brain cortical regions throughout a complex cognitive task. We found a similar functional connectivity pattern in the encoding and the retrieval phases; namely, the positive correlation between left IPL within DMN and left DLPFC within WMN. DLPFC is well known to have a supramodal function, being active in a number of cognitive tasks requiring attention, manipulation and response selection, while IPL has been associated with retrieval and successful recollection [20]. Furthermore, Woodward et al. recently found an involvement of IPL in the encoding phase as well [17]. This suggests the existence of a specific type of cooperation between the two networks taking place during the encoding and retrieval phases in which the DLPFC becomes positively correlated with IPL, at least in the left hemisphere. On the other hand, because the two phases have the presentation of a visual stimulus in common, we cannot rule out that such positive functional connectivity is dynamically induced by the (passive) viewing of the stimulus, independent of the working-memory task. Our MRI acquisition parameters did not allow full coverage of the brain in the given TR (= 1500 ms) for each participant, and therefore failed to provide consistent data from visual cortex across our sample. This absence of a potential control region for our network analyses within the visual cortex that is also activated by the task but does not show the described stage-dependent dynamics in inter-network connectivity represents a limitation of the current study.

We also found a positive correlation between the right parietal regions belonging to both networks in the retrieval phase (Fig 4). From a cognitive perspective, the retrieval phase is characterized by a matching and selection process between the memorized stimulus (from the encoding phase) and the stimulus presented during retrieval. Emerging evidences [16] show a functional dissociation of sub-regions within parietal cortices, with the superior posterior parietal cortex (SPL/IPS) not being directly involved in retrieval but rather in the old/new stimulus selection process, and the IPL relating to the successful recollection of information. Furthermore, Shannon et al. [37] demonstrated that IPL is involved in recollection, irrespective of the type of the stimulus (visual or verbal), and that its activation is not influenced by spatial manipulation of the stimulus. In other words, the role of IPL seems to correspond to the integration of multi-modal information, reflecting Baddeley’s “episodic buffer” [38]. The above mentioned results suggest that a cooperation between IPL and IPS is involved in the retrieval phase of a memory process and our findings of a positive correlation between IPL and IPS respectively in the DMN and WMN would support the hypothesis of a specific functional communication, needed to recognize the novelty of the visual stimulus and recollect previously stored information to select the appropriate response [16,22,37].

The positive correlations between the regions of the two networks are not present during the maintenance phase, when no external stimulus is presented and the information is purely stored, manipulated, and kept in memory. This suggests that during a pure maintenance phase of the working memory process, subjects are focused on the internal representation of the information and need to actively avoid or minimize any external or internal distraction. Therefore, one possible explanation for the shift from positive to negative connection might be that, for successful maintenance of specific information, it is necessary to keep the WMN functionally separated from the DMN, in such a way to keep maintenance operations undisturbed from typical internal cognition processes, such as, e. g., mind wandering [39], autobiographic memory [40] and self-awareness streaming [41].

Previous studies have revealed an activation/deactivation dichotomy even in resting state functional connectivity analyses, showing that the DMN’s signal is anti-correlated with the signal from the task-positive networks [3]. However, we believe that this dichotomous view cannot be automatically extended from the resting to all possible brain and/or cognitive states, such as those arising from the application a cognitive task, from basically two reasons: first, nothing is known about functional connectivity modulation of selected pairs of ROIs during distinct stages of cognitive task. Second, recent papers show a functional heterogeneity of DMN, if determined during a cognitive task. For instance, Mayer et al. [30] suggested that the DMN can be dissociated into subcomponents, some of which have a task dependent specificity, while other components are considered “the core” of the network as they deactivate proportionally to cognitive load but not to a specific task [30]. Furthermore, the posterior cingulate cortex can be divided into two components, ventral and dorsal, showing dissociated functional connectivity relatively to task load: the ventral part showed reduced functional connectivity with the rest of DMN and reduced anti-correlation with the WMN components, while the dorsal part showed the opposite pattern, with increased correlation with the DMN and increased anti-correlation with the WMN [42].

Although our activation results (load dependent activation of WMN and deactivation of DMN) replicated previous findings, our multi-phase functional connectivity approach did not reveal an overall anti-correlation between the two networks across all phases of the task. On the other hand, previous reports of such anti-correlated activity mainly relied on data from resting state or N-back experiments, which are create relatively “stationary” conditions, with or without cognitive engagement. In contrast, our delayed task, combined with the analysis of functional connectivity across different task phases, allowed us to define a dynamic functional connectivity model by disentangling the working memory phases and assign a specific pattern of connectivity to each phase.

In conclusion, our results support previous reports about a direct and active involvement of DMN in working memory, mainly via the interaction of its parietal nodes with prefrontal regions of the WMN, but also show, for the first time, the detailed dynamics of functional connectivity between networks during distinct stages of a cognitive task, thereby giving new insights to the comprehension of human brain functioning. The dichotomous organization of human brain in anti-correlated networks, as revealed by Fox in the resting brain [3], is disrupted when the brain is involved in some cognitive processes. Moreover, functional connectivity among networks is modulated by the complexity of cognitive functions that are often multi-modal and the combination of a series of sub-processes.

These findings open new insights in understanding neural mechanism underlying a cognitive process such as working memory, which seems to need specific and dynamic interactions between two networks. Moreover they suggest that the DMN activity is not specific of resting condition but is actively implicated in human cognition. Because DMN is functionally affected in early stages of some neurological diseases, particularly Alzheimer’s Disease [2], our results are potentially useful in understanding some aspects of cognitive deficit in Alzheimer’s Disease and in developing specific rehabilitative protocols for non invasive brain stimulation, such as Transcranial Direct Current Stimulation (tDCS) or Trancranial Magnetic Stimulation (TMS), which are able to affect both excitability and functional connectivity of brain cortex [43–46].

## Supporting Information

S1 Table Talairach coordinates of peaks in selected ROIs (single subject). PCC/Rsp: posterior cingulate/retrosplenial cortex; MPFC: medial prefrontal cortex; IPL: inferior parietal lobule; IPS: intra-parietal sulcus; DLPFC: dorso-lateral prefrontal cortex.
(DOCX)

S2 Table Talairach coordinates of peaks in auditory cortex (single subject). R-Au: right auditory cortex; L-Au: left auditory cortex.
(DOCX)

S3 Table Modulation of functional connectivity between right and left auditory cortex and all the ROIs. Across-subjects two-way repeated measures ANOVA with load and phase as within subjects factor (p values). Au: auditory cortex; PCC/Rsp: posterior cingulate/retrosplenial cortex; MPFC: medial prefrontal cortex; IPL: inferior parietal lobule; IPS: intra-parietal sulcus; DLPFC: dorso-lateral prefrontal cortex. No p values exceeded FDR threshold (q = 0.05).
(DOCX)
