# Supplementary material for: The Default Mode Network and the Working Memory Network Are Not Anti-Correlated during All Phases of a Working Memory Task
Source: PLoS One. 2015 Apr 7;10(4):e0123354. doi: 10.1371/journal.pone.0123354 (PMC4388669; doi:10.1371/journal.pone.0123354)
Supplement: S1 Table — PCC/Rsp: posterior cingulate/retrosplenial cortex; MPFC: medial prefrontal cortex; IPL: inferior parietal lobule; IPS: intra-parietal sulcus; DLPFC: dorso-lateral prefrontal cortex. (DOCX) [file pone.0123354.s001.docx]

**Table S1**: **Talairach coordinates of peaks in selected ROIs (single subject)**

| **SUBJECTS** | **REGIONS** | **COORDINATES** | | | **SUBJECTS** | **REGIONS** | **COORDINATES** | | | **SUBJECTS** | | **REGIONS** | | **COORDINATES** | | | |  |
| --- | --- | --- | --- | --- | --- | --- | --- | --- | --- | --- | --- | --- | --- | --- | --- | --- | --- | --- |
|  |  | **X** | **Y** | **Z** |  |  | **X** | **Y** | **Z** |  |  | |  | | **X** | **Y** | **Z** | |
| ♯1 | **PCC/Rsp** | 2 | -44 | 31 | ♯6 | **PCC/Rsp** | -3 | -48 | 26 |  | ♯11 | | **PCC/Rsp** | | -9 | -51 | 26 | |
|  | **MPFC** | -2 | 25 | -8 |  | **MPFC** | -3 | 41 | 30 |  |  | | **MPFC** | | -2 | 31 | -4 | |
|  | **Right IPL** | 41 | -56 | 33 |  | **Right IPL** | 30 | -81 | 36 |  |  | | **Right IPL** | | 53 | -64 | 21 | |
|  | **Left IPL** | -54 | -63 | 20 |  | **Left IPL** | -43 | -74 | 32 |  |  | | **Left IPL** | | -46 | -75 | 24 | |
|  | **Right IPS** | 28 | -56 | 47 |  | **Right IPS** | 44 | -32 | 36 |  |  | | **Right IPS** | | 26 | -66 | 36 | |
|  | **Left IPS** | -25 | -59 | 45 |  | **Left IPS** | -36 | -54 | 44 |  |  | | **Left IPS** | | -37 | -37 | 38 | |
|  | **Right DLPFC** | 44 | 3 | 24 |  | **Right DLPFC** | 42 | 38 | 39 |  |  | | **Right DLPFC** | | 34 | 19 | 36 | |
|  | **Left DLPFC** | -55 | 5 | 30 |  | **Left DLPFC** | -41 | 30 | 38 |  |  | | **Left DLPFC** | | -31 | 19 | 26 | |
| ♯2 | **PCC/Rsp** | -2 | -48 | 24 | ♯7 | **PCC/Rsp** | -1 | -40 | 28 |  | ♯12 | | **PCC/Rsp** | | -10 | -55 | 32 | |
|  | **MPFC** | 2 | 46 | 9 |  | **MPFC** | 1 | 29 | -6 |  |  | | **MPFC** | | -4 | 30 | -5 | |
|  | **Right IPL** | 50 | -59 | 23 |  | **Right IPL** | 41 | -77 | 38 |  |  | | **Right IPL** | | 39 | -71 | 34 | |
|  | **Left IPL** | -44 | -71 | 30 |  | **Left IPL** | -50 | -74 | 20 |  |  | | **Left IPL** | | -48 | -77 | 30 | |
|  | **Right IPS** | 35 | -58 | 50 |  | **Right IPS** | 23 | -67 | 48 |  |  | | **Right IPS** | | 31 | -44 | 37 | |
|  | **Left IPS** | -18 | -70 | 46 |  | **Left IPS** | -34 | -62 | 42 |  |  | | **Left IPS** | | -31 | -50 | 33 | |
|  | **Right DLPFC** | 40 | 18 | 35 |  | **Right DLPFC** | 35 | 29 | 25 |  |  | | **Right DLPFC** | | 51 | 28 | 32 | |
|  | **Left DLPFC** | -27 | 31 | 28 |  | **Left DLPFC** | -21 | 35 | 28 |  |  | | **Left DLPFC** | | -49 | 25 | 33 | |
| ♯3 | **PCC/Rsp** | -10 | -53 | 13 | ♯6 | **PCC/Rsp** | -3 | -54 | 20 |  | ♯13 | | **PCC/Rsp** | | -8 | -35 | 33 | |
|  | **MPFC** | 2 | 46 | 21 |  | **MPFC** | 4 | 52 | 11 |  |  | | **MPFC** | | 10 | 34 | 5 | |
|  | **Right IPL** | 46 | -70 | 26 |  | **Right IPL** | 56 | -58 | 32 |  |  | | **Right IPL** | | 50 | -65 | 30 | |
|  | **Left IPL** | -50 | -65 | 38 |  | **Left IPL** | -53 | -58 | 21 |  |  | | **Left IPL** | | -54 | -65 | 28 | |
|  | **Right IPS** | 40 | -53 | 48 |  | **Right IPS** | 24 | -50 | 47 |  |  | | **Right IPS** | | 37 | -74 | 35 | |
|  | **Left IPS** | -33 | -53 | 47 |  | **Left IPS** | -29 | -50 | 41 |  |  | | **Left IPS** | | -31 | -71 | 39 | |
|  | **Right DLPFC** | 43 | 22 | 27 |  | **Right DLPFC** | 22 | 28 | 28 |  |  | | **Right DLPFC** | | 35 | 36 | 38 | |
|  | **Left DLPFC** | -38 | 16 | 24 |  | **Left DLPFC** | -36 | 25 | 26 |  |  | | **Left DLPFC** | | -45 | 32 | 31 | |
| ♯4 | **PCC/Rsp** | -4 | -37 | 36 | ♯9 | **PCC/Rsp** | 0 | -50 | 31 |  | ♯14 | | **PCC/Rsp** | | 8 | -47 | 30 | |
|  | **MPFC** | -3 | 28 | -3 |  | **MPFC** | -1 | 27 | -13 |  |  | | **MPFC** | | 2 | 42 | 2 | |
|  | **Right IPL** | 58 | -53 | 24 |  | **Right IPL** | 54 | -63 | 24 |  |  | | **Right IPL** | | 44 | -74 | 22 | |
|  | **Left IPL** | -55 | -64 | 25 |  | **Left IPL** | -46 | -65 | 26 |  |  | | **Left IPL** | | -48 | -71 | 24 | |
|  | **Right IPS** | 33 | -56 | 44 |  | **Right IPS** | 17 | -66 | 54 |  |  | | **Right IPS** | | 26 | -68 | 45 | |
|  | **Left IPS** | -36 | -38 | 37 |  | **Left IPS** | -22 | -64 | 51 |  |  | | **Left IPS** | | -43 | -38 | 45 | |
|  | **Right DLPFC** | 35 | 21 | 38 |  | **Right DLPFC** | 44 | 35 | 27 |  |  | | **Right DLPFC** | | 38 | 34 | 30 | |
|  | **Left DLPFC** | -45 | 22 | 32 |  | **Left DLPFC** | -43 | 24 | 33 |  |  | | **Left DLPFC** | | -40 | 40 | 24 | |
| ♯5 | **PCC/Rsp** | 2 | -44 | 30 | ♯10 | **PCC/Rsp** | -3 | -42 | 29 |  |  | |  | |  |  |  | |
|  | **MPFC** | -7 | 37 | 9 |  | **MPFC** | 0 | 37 | -3 |  |  | |  | |  |  |  | |
|  | **Right IPL** | 59 | -29 | 24 |  | **Right IPL** | 39 | -69 | 28 |  |  | |  | |  |  |  | |
|  | **Left IPL** | -61 | -38 | 15 |  | **Left IPL** | -52 | -70 | 24 |  |  | |  | |  |  |  | |
|  | **Right IPS** | 30 | -53 | 30 |  | **Right IPS** | 30 | -57 | 39 |  |  | |  | |  |  |  | |
|  | **Left IPS** | -40 | -50 | 33 |  | **Left IPS** | -38 | -44 | 36 |  |  | |  | |  |  |  | |
|  | **Right DLPFC** | 32 | 43 | 28 |  | **Right DLPFC** | 44 | 30 | 33 |  |  | |  | |  |  |  | |
|  | **Left DLPFC** | -55 | 16 | 33 |  | **Left DLPFC** | -28 | 28 | 33 |  |  | |  | |  |  |  | |

PCC/Rsp: posterior cingulate/retrosplenial cortex; MPFC: medial prefrontal cortex; IPL: inferior parietal lobule; IPS: intra-parietal sulcus; DLPFC: dorso-lateral prefrontal cortex.
