# Supplementary material for: The Default Mode Network and the Working Memory Network Are Not Anti-Correlated during All Phases of a Working Memory Task
Source: PLoS One. 2015 Apr 7;10(4):e0123354. doi: 10.1371/journal.pone.0123354 (PMC4388669; doi:10.1371/journal.pone.0123354)
Supplement: S2 Table — R-Au: right auditory cortex; L-Au: left auditory cortex. (DOCX) [file pone.0123354.s002.docx]

**Table S2: Talairach coordinates of peaks in auditory cortex (single subject)**

| **SUBJECTS** | **REGIONS** | **COORDINATES** | | |
| --- | --- | --- | --- | --- |
|  |  | **X** | **Y** | **Z** |
| ♯1 | **R-Au** | -55 | -23 | 21 |
|  | **L-Au** | 50 | -23 | 21 |
| ♯2 | **R-Au** | 41 | -35 | 15 |
|  | **L-Au** | -55 | -23 | 21 |
| ♯3 | **R-Au** | 47 | -26 | 9 |
|  | **L-Au** | -52 | -17 | 12 |
| ♯4 | **R-Au** | 44 | -29 | 16 |
|  | **L-Au** | -52 | -32 | 15 |
| ♯5 | **R-Au** | 69 | -20 | 12 |
|  | **L-Au** | -46 | -23 | 12 |
| ♯6 | **R-Au** | 42 | -19 | 15 |
|  | **L-Au** | -43 | -29 | 12 |
| ♯7 | **R-Au** | 53 | -24 | 6 |
|  | **L-Au** | -68 | -29 | 12 |
| ♯8 | **R-Au** | 50 | -23 | 15 |
|  | **L-Au** | -55 | -29 | 12 |
| ♯9 | **R-Au** | 47 | -23 | 15 |
|  | **L-Au** | -52 | -20 | 12 |
| ♯10 | **R-Au** | 66 | -17 | 15 |
|  | **L-Au** | -46 | -17 | 12 |
| ♯11 | **R-Au** | 44 | -23 | 12 |
|  | **L-Au** | -52 | -35 | 9 |
| ♯12 | **R-Au** | 41 | -20 | 9 |
|  | **L-Au** | -52 | -26 | 9 |
| ♯13 | **R-Au** | 53 | -29 | 16 |
|  | **L-Au** | -58 | -23 | 9 |
| ♯14 | **R-Au** | -51 | -17 | 16 |
|  | **L-Au** | 44 | -20 | 6 |

R-Au: right auditory cortex; L-Au: left auditory cortex.
