# Supplementary material for: The Default Mode Network and the Working Memory Network Are Not Anti-Correlated during All Phases of a Working Memory Task
Source: PLoS One. 2015 Apr 7;10(4):e0123354. doi: 10.1371/journal.pone.0123354 (PMC4388669; doi:10.1371/journal.pone.0123354)
Supplement: S3 Table — Across-subjects two-way repeated measures ANOVA with load and phase as within subjects factor (p values). Au: auditory cortex; PCC/Rsp: posterior cingulate/retrosplenial cortex; MPFC: medial prefrontal cortex; IPL: inferior parietal lobule; IPS: intra-parietal sulcus; DLPFC: dorso-lateral prefrontal cortex. No p values exceeded FDR threshold (q = 0.05). (DOCX) [file pone.0123354.s003.docx]

Table S3: modulation of functional connectivity between right and left auditory cortex and all the ROIs. Across-subjects two-way repeated measures ANOVA with load and phase as within subjects factor (p values).

| **PAIRS** | **PHASE** | **LOAD** | **INTERACTIONS** |
| --- | --- | --- | --- |
| *Auditory cortex vs*  *Default Mode Network* | p | p | p |
| **Right Au – PCC/Rsp** | 0.7868 | 0.3177 | 0.0917 |
| **Right Au – MPFC** | 0.0628 | 0.0122 | 0.9219 |
| **Right Au - Right IPL** | 0.4014 | 0.5930 | 0.5962 |
| **Right Au - Left IPL** | 0.9257 | 0.3548 | 0.3471 |
| **Left Au – PCC/Rsp** | 0.0205 | 0.5392 | 0.5622 |
| **Left Au - MPFC** | 0.0334 | 0.0493 | 0.8996 |
| **Left Au - Right IPL** | 0.3554 | 0.2617 | 0.1041 |
| **Left Au - Left IPL** | 0.2181 | 0.0895 | 0.1781 |
|  |  |  |  |
| *Auditory cortex vs*  *Working Memory Network* |  |  |  |
| **Right Au – Right IPS** | 0.0510 | 0.5234 | 0.7713 |
| **Right Au – Left IPS** | 0.1561 | 0.3632 | 0.4403 |
| **Right Au – Right DLPFC** | 0.0297 | 0.7638 | 0.4131 |
| **Right Au – Left DLPFC** | 0.0545 | 0.5145 | 0.4169 |
| **Left Au – Right IPS** | 0.2322 | 0.6147 | 0.6930 |
| **Left Au – Left IPS** | 0.5606 | 0.5557 | 0.9028 |
| **Left Au – Right DLPFC** | 0.6093 | 0.4287 | 0.3519 |
| **Left Au – Left DLPFC** | 0.1688 | 0.0433 | 0.9343 |

Au: auditory cortex; PCC/Rsp: posterior cingulate/retrosplenial cortex; MPFC: medial prefrontal cortex; IPL: inferior parietal lobule; IPS: intra-parietal sulcus; DLPFC: dorso-lateral prefrontal cortex.

No p values exceeded FDR threshold (q = 0.05).
